# Symptoms of bipolar disorder among adolescents residing at Youth Educational Centers in Silesia in Poland

**DOI:** 10.1192/j.eurpsy.2023.1524

**Published:** 2023-07-19

**Authors:** K. Kamińska, M. A. Ciołek, I. Rosół, M. Potaczek, M. Matlakiewicz, K. Wilczyński, M. Janas-Kozik

**Affiliations:** 1 Students’ scientific association at the Department of Psychiatry and Psychotherapy of Developmental Age; 2 Department of Psychiatry and Psychotherapy of Developmental Age, Medical University of Silesia, Katowice; 3 Pediatric Centre of John Paul II, Sosnowiec, Poland

## Abstract

**Introduction:**

According to the Journal of Laws of the Republic of Poland, juveniles considered to be socially maladjusted are referred to the facilities called Youth Educational Centres. The aforementioned decision must be processed by court. Adolescents held there are guilty mainly of truancy, acts of vandalism, and the crime of theft. Other reasons might include substance abuse, fleeing from home and loitering without legal guardian’s supervision. The key purpose behind those institutions is adjusting to social standards, as well as rehabilitation of each juvenile delinquent. On the other hand, such behaviors may result from various mental disorders, which are often overlooked and underestimated.

**Objectives:**

The aim of this study was to assess the prevalence of psychiatric disorders among the inmates of Youth Educational Centres with a primary Focus on bipolar disorder. The existing knowledge on this subject is insufficient whereas the only available papers are based on retrospective studies of documents, which may have led to underestimation of mental condition in this population.

**Methods:**

Patients were examined in person, using the K-SADS diagnostic interview. The missing details were also collected during reviewing the inmates’ documents. The study group consists of juveniles staying in two Youth Educational Centres, a male and female one, located in Silesian Province of southern Poland. Participation in the research was voluntary whereas information gathered during the interview remains confidential.

**Results:**

The study included 80 adolescents who previously had consented to participate. 60% (n=48) of them were males. Among the male patients 27.08% met the criteria for an episode of mania/hypomania, 37.5% for a depressive disorder whereas 22.92% fulfilled the criteria for both mania and depression. Seven boys were diagnosed beforehand, those included: one case of bipolar disorder, one schizoaffective disorder and five of them were receiving outpatient treatment for depressive episodes. Among female inmates: 40.63% met the criteria for both mania/hypomania and depressive disorder whereas as many as 78.13% claimed to have depressive disorder. Two girls have already been diagnosed – one suffered from bipolar disorder, manic depression and schizophrenia and the other was treated for depressive episodes.

**Conclusions:**

Social maladjustment is often accompanied by a mental disorder or may be caused by one. Psychiatric disorders in adolescents, particularly bipolar disorder, usually have an atypical course, which can delay the appropriate diagnosis. Postponement of the crucial treatment is directly related to significant deterioration of the patient’s prognosis. In order to provide adequate and necessary support, juveniles referred to the Youth Educational Centres should be examined by a certified psychiatrist before the admission to such facility.

**Disclosure of Interest:**

None Declared

